# Interdependent = Compassionate? Compassionate and Self-Image Goals and Their Relationships With Interdependence in the United States and Japan

**DOI:** 10.3389/fpsyg.2019.00192

**Published:** 2019-02-07

**Authors:** Yu Niiya, Jennifer Crocker

**Affiliations:** ^1^Department of Global and Interdisciplinary Studies, Hosei University, Tokyo, Japan; ^2^Department of Psychology, Ohio State University, Columbus, OH, United States

**Keywords:** compassionate goals, self-image goals, interdependence, United States, Japan, measurement invariance, scale

## Abstract

The pursuit of compassionate goals, which involves focusing on and attending to other people’s needs, has often been described as a defining characteristic of an interdependent self that prioritizes harmonious relationships over individual achievement. This research investigated whether compassionate goals can be empirically distinguished from various indices of interdependence and examined their correlations with interdependence in both American and Japanese adult samples. It further differentiated two types of self-image goals—the goals to appear warm and kind vs. the goals to appear competent and in control—and explored their relationships with interdependence. In Study 1, the 18-item scale showed a clear four-factor structure that distinguished (a) compassionate goals, (b) approach-worded likable self-image goals, (c) approach-worded competent self-image goals, and (d) avoidance-worded self-image goals. Study 2 confirmed the equivalence of the four-factor structure and the equivalence of factor loadings in the United States and Japan. Finally, Study 3 showed that the items of compassionate goals and those of various measures of interdependence loaded onto separate factors with only negligible cross-loadings. Study 3 further found that the indices of interdependence reflecting connection with others showed moderately positive correlations with compassionate goals whereas indices of interdependence reflecting conformity showed moderate correlations with likable, competent, and avoidant self-image goals, indicating that the pursuit of compassionate and self-image goals reflect different aspects of interdependence.

## Introduction

Culture shapes the ideal self and the goals people pursue. In an independent cultural context, people view themselves as a bounded entity characterized by internal attributes such as preferences, abilities, and traits, and seek to maintain their self-view as an independent, autonomous, and competent agent (Markus and Kitayama, 1994). In contrast, in an interdependent cultural context, people view themselves as inherently connected with others, seek to adjust and fit in to important relationships and groups, and prioritize collective over personal goals (Markus and Kitayama, 1994). Although interdependence is generally more prevalent in East Asian countries, such as Japan, and independence in North American and Western European countries, such as the United States, (Kitayama et al., 2009; Na et al., 2010; Vignoles et al., 2016), research has also shown variations within a single culture (Markus and Conner, 2013; Markus, 2017). For example, even within the United States, racial minorities are more interdependent than Whites and those from the working class are more interdependent than those from the middle class. In an independent culture, people strive to appear competent whereas in an interdependent culture, people strive to appear likable, because possessing these ideal characteristics usually results in greater acceptance and better social outcomes (Oishi and Diener, 2001; Kitayama and Cohen, 2007; Chen and Jing, 2012).

Possessing the ideal characteristics of a culture may be beneficial, but striving to *appear* as possessing desirable characteristics may be more problematic. People who pursue the goals to project a desirable self-image to others (i.e., self-image goals) to obtain approval, recognition, or love, ironically experience decreased acceptance (Canevello and Crocker, 2011), decreased relationship quality (Canevello and Crocker, 2010), increased anxiety, dysphoria and distress (Crocker et al., 2010) and increased relationship insecurity over time (Canevello et al., 2013). Rather than promoting a self-image of competence or warmth, people can pursue goals to support others’ well-being (i.e., compassionate goals) and build fulfilling relationships with others. People with compassionate goals are more responsiveness to others’ needs, receive higher esteem from others (Canevello and Crocker, 2011), and have more satisfying relationships over time (Crocker and Canevello, 2008; Canevello and Crocker, 2010; Canevello et al., 2013; Crocker et al., 2017). Cumulative evidence attests to the importance of compassionate goals in fostering relationships and well-being, but to date, very little research has investigated the cultural underpinning of compassionate goals. What cultures breed compassionate goals? Are compassionate goals a characteristic of interdependence?

The pursuit of compassionate goals, which involves focusing on and attending to other people’s needs, might seem to be a defining characteristic of an interdependent self. People with interdependent selves include other people and relationships as part of their mental representations of themselves and prioritize harmonious relationships over individual achievement (Fiske et al., 1998). Indeed, people who have compassionate goals have more interdependent self-construals, and develop increasingly interdependent self-construals over time (Jiang et al., 2017). Thus, compassionate goals and interdependent self-construals may be highly overlapping, and even, one might argue, conceptually indistinguishable.

We propose, however, that interdependent selves and compassionate goals are related but distinct constructs. The pursuit of compassionate goals is only one of the many forms that interdependence can take. Moreover, any behavior that reflects interdependence may result from either compassionate or self-oriented goals, or both. For example, interdependence can be manifested as having the desire to fit in with others or sacrificing personal goals for others (Markus and Kitayama, 1991). People may conform to others’ decisions to support the group’s mission (i.e., compassionate goals) but they may also do so to avoid being seen as a nuisance to others (i.e., self-image goals). Thus, interdependence may be associated with both compassionate goals and self-image goals, and in particular with the goal to appear likable.

The present research investigates the overlap between interdependence and compassionate goals by examining whether compassionate goals can be empirically distinguished from various indices of interdependence and by examining how they are associated with each other in both American and Japanese samples. It further differentiates two types of self-image goals—the goals to appear warm and kind vs. the goals to appear competent and in control—and explores their relationships with interdependence. To this end, we present a new measure of compassionate and self-image goals and assess its measurement invariance across the United States and Japan.

### Are Compassionate Goals a Defining Feature of an Interdependent Self?

People with interdependent self-construals define themselves by social roles, relationships, norms, and contexts (Fiske et al., 1998) and place value in maintaining harmonious relationships with others, meeting social obligations and expectations, adjusting and fitting in to important relationships, and promoting the goals of others (Markus and Kitayama, 1991, 1994). In the interdependent mode of the self, people are “expected to take the perspective of others in the relationship, feel empathically with them, and act accordingly, often altruistically, on others’ behalf (Kitayama and Markus, 2000, p. 120).” Indeed, compassion and sympathy have often been described as characteristics of interdependent self-construal and collectivism in both the United States and Japan (Uchida and Kitayama, 2001; Dalsky et al., 2008). If compassion and sympathy are the focal mechanism through which people maintain interdependence (Kitayama and Markus, 2000), and if other-profitable characteristics such as being helpful and loving are quintessential collectivistic values (Wojciszke, 1997), then it seems that compassionate goals should be a defining feature of interdependence and that compassionate goals and interdependence may be conceptually indistinguishable.

Despite some conceptual overlap between interdependence and compassionate goals, one can also argue that the pursuit of compassionate goals is only one of the many forms that interdependence can take. Markus and Kitayama (1991) described different aspects of interdependence, including viewing oneself as connected to others, fitting in with others, sacrificing personal goals for others, and exercising self-restraint. In a large cross-cultural study, Vignoles et al. (2016) identified seven dimensions of interdependence, including dependence on others, connection to others, similarity with others, commitment to others, variability of the self across situations, receptiveness to influence, and valuing group harmony. Although some of these aspects (e.g., connection, commitment, and sacrificing personal goals for others) should relate to compassionate goals, other aspects (e.g., dependence, similarity, and receptiveness to influence) may be independent of compassionate goals. Moreover, even when some aspects of interdependence correlate more strongly with compassionate goals, the underlying reasons for adopting an interdependent behavior may or may not stem from compassionate goals. For example, a person who acts differently in different social environments or a person who hides his inner feelings to adapt to the surrounding people shows the characteristics of an interdependent self but the individual may not necessarily do so out of compassion for others. One may adjust to others or maintain group harmony to support others’ well-being (compassionate goals), to avoid being ostracized (likable self-image goals), or to show that they are an excellent member of the group (competent self-image goals). As a result, interdependence should be only moderately positively correlated with compassionate goals.

Consistent with this reasoning, compassionate goals correlate only moderately with interdependence. Compassionate goals were positively and moderately correlated with interdependence as measured by a modified Singelis scale in Japanese undergraduate (0.38) and adult samples (0.32), and not correlated among American undergraduates (-0.08; Niiya et al., 2013). Compassionate goals also showed a weak positive correlation (0.11) with interdependence in Poland (Kuncewicz et al., 2015). The Japanese compassionate goals scale (Niiya, 2016) showed a moderate positive correlation with Takata’s (2000) measure of interdependence (0.31), a slightly stronger correlation with the affinity for others subscale (0.42) and a weaker positive correlation with the evaluation apprehension subscale (0.13). In the United States, compassionate goals correlated positively with relational interdependent self-construal (i.e., the extent to which people define themselves in terms of their close personal relationships; Cross et al., 2000) among undergraduates (0.41) and romantic couples (0.23) and compassionate goals predicted increased relational interdependent self-construal over 3 weeks and 10 weeks (Jiang et al., 2017). Taken together, these findings suggest that compassionate goals are empirically related but not necessarily a core component of interdependence. Therefore, the present study tests the hypothesis that compassionate goals can be conceptually and empirically distinguished from various measures of interdependence in both Americans and Japanese.

### Are People With an Interdependent Self More Likely to Pursue Self-Image Goals?

Because “fitting in” is an important goal for those with an interdependent self (Markus and Kitayama, 2010), people with an interdependent self might seek acceptance from others by showing desirable aspects of themselves; that is, they might have self-image goals. However, support for this idea is mixed: Interdependence, as measured by a modified Singelis scale, was positively but only weakly correlated with self-image goals among Japanese undergraduates (0.14) and adults (0.19; Niiya et al., 2013), and not significantly correlated among American (0.08; Niiya et al., 2013) and Polish undergraduates (-0.01; Kuncewicz et al., 2015). In contrast, the Japanese self-image goals scale (Niiya, 2016) showed a stronger positive correlation with both subscales of Takata’s (2000) interdependence (0.54 with affinity for others and 0.51 with evaluation apprehension) among the Japanese adults.

One possible explanation for these mixed findings is that the content of self-image goals differed between the original English scale (Crocker and Canevello, 2012) and the revised Japanese scale (Niiya, 2016). Niiya et al. (2013) first translated the 12-item compassionate and self-image goals scale used in Crocker and Canevello (2012) into Japanese, but to achieve a good model fit, four items had to be excluded, which reduced the number of items to four each and consequently reduced their reliabilities (0.68–0.72 for compassionate goals and 0.55–0.62 for self-image goals in Niiya et al., 2013). To increase the number of items and to address the possibility that desirable self-images vary by culture (Chen and Jing, 2012), Niiya (2016) created a Japanese version of the Compassionate and Self-Image Goals scale, adding self-image goals items that reflect the goals to promote a warm and likable impression of the self (e.g., “be seen as a kind person”) or avoid promoting an unlikable impression of the self (e.g., “avoid being disliked”), a self-image that was considered more important to those with a collectivistic than an individualistic value orientation (Wojciszke, 1997; Chen and Jing, 2012). The resulting scale, comprising 11 items each for compassionate and self-image goals, had a clear two-factor structure and showed excellent reliabilities (0.90 for compassionate goals and 0.93 for self-image goals). However, the self-image goals items in the revised Japanese scale contrasted with the original self-image goals measure by Crocker and Canevello (2012), which comprised six items that did not distinguish competent and likable self-image goals (e.g., “get others to recognize or acknowledge your positive qualities” or “convince others that you are right”).

Distinguishing the goal to appear likable from the goal to appear competent is essential in understanding human interactions. Warmth (likability) and competence are the “Big Two” fundamental and universal dimensions that underlie judgments of the self and others (Rosenberg et al., 1968; Fiske et al., 2007; Abele and Wojciszke, 2013). Warmth (likability) and competence are also two impressions that people try to promote in interactions. The literature on self-presentation has distinguished two forms of impression management: self-promoting goals that seek to enhance perceived competence and ingratiation goals that seek to enhance likability (e.g., Godfrey et al., 1986; DePaulo et al., 1987; Feldman et al., 2002). Distinguishing competent from likable self-image goals would allow researchers to test whether the content of self-image goals moderates the negative influence of these goals on relationship quality and well-being.

The distinction between likable and competent self-image goals is especially important in cultural research, given the frequently reported contrast between Western individualistic societies that value competence and autonomy and East Asian collectivistic societies that value interpersonal warmth and communality (Judd et al., 2005). Individualistic values, such as being independent, intellectual, and capable were rated as being more related to competence than morality (warmth); in contrast, collectivistic values, such as being loving and polite were rated as being more related to morality (warmth) than competence (Wojciszke, 1997). Because warmth is a more collectivistic value and competence a more individualistic value, interdependence may correlate more strongly with likable self-image goals than with competent self-image goals, which may explain the mixed correlations in previous research.

Recently, Canevello and Crocker (unpublished) developed an unpublished scale that distinguished compassionate goals, likable self-image goals, and competent self-image goals in the United States but no research to date has attempted to measure them cross-culturally. The current research aimed to devise a compassionate and self-image goals scale that distinguishes competent and likable self-image goals and shows measurement invariance in the United States and Japan. We then examined their correlations with various measures of interdependence.

## Study 1

Studies 1 and 2 aimed to develop a compassionate and self-image goals scale that shows measurement invariance across the United States and Japan. In Study 1, a series of exploratory factor analyses (EFAs) were performed in a combined sample of American and Japanese adults to select compassionate and self-image goals items with a clear and meaningful factor structure.

### Methods

#### Participants

For the American sample, 719 participants who were currently living in the United States and who were 18 years or older were recruited through Amazon’s MTurk. From this initial sample, 49 were excluded for having spent less than 2 s per question, 26 for failing the attention check question (“please select the second option”), and 75 for having a variance of 0 on at least one of the five pages containing the goals items (10 items were displayed on each page). The final sample consisted of 569 participants (46.7% female) with a mean age of 36.37 (*SD* = 11.12), ranging from 20 to 79. The large majority (94.9%) reported having lived in the United States for their entire life. Most (67.7%) had all four grandparents born in the United States, 23.3% had one, two, or three grandparents born in the United States, and 9.0% had no grandparents born in the United States. Only 13 (2.3%) reported having lived in Japan but except for one participant who indicated having lived there for 26 years, none had lived in Japan more than 6 years.^1^ The majority (79.3%) were European or White, 12.0% African American or Black, 4.7% Asian, 4.6% Latino or Hispanic, and 4.4% indicated other ethnicities.

In Japan, 2409 participants were recruited through a web survey company (Marketing Applications Inc.). Of these, 54 who had skipped an entire page and 625 who had a variance of zero on at least one of the five pages (which displayed 10 items each) were excluded, leaving a sample of 1714 participants. To match the number of participants in the United States, a randomly selected 580 participants were included in EFAs in Study 1 and the remaining 1134 were included in confirmatory factor analyses in Study 2. The 580 Japanese participants in Study 1 had a mean age of 36.84 (*SD* = 12.10), ranging in age from 20 to 76, and consisted of 50.9% women. In the United States, the informed consent form was displayed on the first page of the survey. In Japan, the informed consent form was displayed in the e-mail sent to recruit participants. In both countries, participants were instructed to start the survey should they agree to participate.

#### Measures

After reading the consent form, participants clicked on the survey link that displayed 50 goals items in random order and some demographic questions. They were asked: “In your everyday relationships, how much do you want or try to do the following?” and rated each item on a scale from 1 = *not at all* to 5 = *very much*. Twenty-eight items from Canevello and Crocker (unpublished), two items from Jiang et al. (2017), and 22 items from Niiya (2016) were aggregated into a scale. Two items overlapped, resulting in a pool of 50 items. The English items were translated into Japanese and the Japanese items were translated into English using the back-translation method. Items are shown in Table 1.

**Table 1:** Summary of exploratory factor analysis results for compassionate and self-image goals measure using principal axis factoring and promax rotation with combined American and Japanese samples in study 1 (*N* = 1149).

	Factor			
	
Item	1	2	3	*h*^2^
Understand the other person’s feelings.^a∗^	**0.77**			0.55
Consider things from the other person’s perspective.^a^	**0.73**			0.46
Have compassion for others’ mistakes and weaknesses.^∗^	**0.72**			0.47
Engage sincerely with others.^a^	**0.69**	–0.22		0.49
Avoid doing anything that would be harmful to others.^b∗^	**0.68**		–0.22	0.45
Be supportive of others.^∗^	**0.67**	–0.24	0.27	0.58
Do things that are helpful to both you and others.	**0.61**			0.49
Be useful to others.^a^	**0.58**		0.22	0.47
Avoid taking away others’ opportunity for growth.^a∗^	**0.58**			0.33
Be constructive in your comments to others.^b^	**0.55**			0.29
Understand how your actions affect others.	**0.54**		0.21	0.39
Avoid neglecting your relationship with others.^∗^	**0.53**			0.34
Avoid hasty judgment.^a^	**0.49**			0.25
Avoid imposing my opinion on others.^a^	**0.47**	0.31	–0.36	0.28
Avoid being caught up in my own prejudice.^a^	**0.46**	0.24		0.29
Be seen as an attentive person.^a^	**0.38**		0.37	0.42
Avoid doing things that aren’t helpful to you or others.	**0.37**	0.20		0.29
Avoid being disliked.^a∗^		**0.70**		0.56
Avoid being embarrassed.^a∗^		**0.69**		0.45
Avoid looking like a failure.^∗^		**0.67**		0.49
Avoid showing your unlikable side.^∗^		**0.64**		0.45
Avoid being exposed as wrong.^∗^	–0.22	**0.64**		0.43
Conceal your past failures.	–0.25	**0.62**		0.39
Avoid showing your weaknesses.^∗^		**0.59**		0.40
Avoid making others think you’re a bad person.		**0.59**		0.53
Avoid revealing your shortcomings or vulnerabilities.		**0.58**	0.22	0.45
Avoid appearing unlikable.	0.20	**0.56**		0.47
Prove that you’re not a jerk.		**0.54**		0.45
Avoid doing things that would annoy others.^a^	0.46	**0.51**	–0.36	0.43
Avoid appearing egotistical.	0.39	**0.43**		0.35
Try not to appear uncaring.	0.31	**0.43**		0.34
Convince others that you are right.^a^	–0.21	**0.42**		0.24
Avoid being seen as a lazy person.^a^		**0.37**	0.26	0.35
Earn respect of others.^a^			**0.77**	0.57
Prove your competence to others.^∗^			**0.72**	0.52
Appear successful.^∗^	–0.21		**0.70**	0.49
Demonstrate my positive qualities. ^c^	0.21		**0.63**	0.47
Get others to recognize or acknowledge your intelligence.^∗^	–0.25	0.21	**0.62**	0.45
Seem like you know what you’re doing.			**0.61**	0.46
Get others to respect or admire me.^c^		0.24	**0.60**	0.50
Be seen as a reliable person.^a^	0.28		**0.60**	0.52
Get others to notice your positive qualities.^a^		0.23	**0.59**	0.54
Make a positive difference in someone else’s life.	0.49	–0.30	**0.50**	0.59
Give the appearance of being on top of things.		0.29	**0.50**	0.47
Do things that you knew you could succeed at			**0.47**	0.32
Get others to think that you are nice.^a∗^		0.36	**0.45**	0.51
Get others to think that you are kind.^a∗^		0.37	**0.43**	0.52
Be seen as a considerate person.^a^	0.40		**0.43**	0.51
Get others to like you.^a^		0.38	**0.42**	0.49
Be seen as a kind person.^a∗^	0.39		**0.41**	0.50
Eigenvalues	14.69	5.57	3.27	
% of variance	29.38	11.14	6.53	

*Factor loadings under 0.20 are suppressed. Bold numbers indicate highest loadings. ^*a*^Items from Niiya (2016). ^*b*^Items that appeared in both Niiya (2016) and Canevello and Crocker (unpublished). ^*c*^Items from Jiang et al. (2017). ^∗^are items that were selected in the 18-item scale.*

### Results and Discussion

An exploratory factor analysis with principal axis factoring for extraction and promax rotation was performed on the combined American and Japanese sample (*n* = 1149). The decrement in eigen values suggested a three-factor solution (14.69, 5.57, 3.27, 1.63, 1.38, etc) that explained 47.1% of total variance. The first factor consisted of 16 compassionate goals items and one likable self-image goals item (“be seen as an attentive person”); the second factor consisted of 15 self-image goals items that were worded as avoiding an undesirable image (both likable and competent); the third factor included 17 self-image goals items worded as approaching a desirable image (both likable and competent) and one compassionate goals item with approach wording (“Make a positive difference in someone else’s life”). The third factor (approach-worded self-image goals) was correlated with both the first factor (compassionate goals; *r* = 0.43) and the second factor (avoidance-worded self-image goals; *r* = 0.44), while the latter two factors were only moderately correlated (*r* = 0.27).

When a two-factor solution was specified, the first factor comprised 26 self-image goals items (both approach and avoidance-worded, likable and competent) and the second factor consisted of all the compassionate goals items and five approach-worded likable self-image goals items and one competent self-image goals item. The two factors explained 40.5% of variance and were positively correlated (*r* = 0.46).

These two analyses suggest that (a) Americans and Japanese generally distinguish compassionate goals from competent and avoidance-worded self-image goals, (b) that they have more difficulty discerning differences between compassionate goals and approach-worded likable self-image goals, and (c) that they distinguish the approach vs. avoidance dimension of self-image goals more than they distinguish the likable vs. competent dimension.

Because the aim of this research was to create a short scale that captures compassionate goals, likable self-image goals, and competent self-image goals, both in terms of approach and avoidance, three items were selected for each category (i.e., approach-worded compassionate goals, avoidance-worded compassionate goals, approach-worded likable self-image goals, avoidance-worded likable self-image goals, approach-worded competent self-image goals, and avoidance-worded competent self-image goals). Items were selected based on the factor loadings from the first EFA and on how well they reflected each concept (see Table 2 for the list of items).^2^ An EFA with the combined American and Japanese sample suggested either a three- or a four-factor solution, with eigen values of 5.72, 2.80, 1.60, 0.96. A three-factor solution, which explained 56.2% of variance, showed a factor structure that resembled the first EFA: the first factor consisted of all the approach and avoidance-worded compassionate goals and one approach-worded likable self-image goals (“be seen as a kind person”). The second factor consisted of six avoidance-worded self-image goals (both likable and competent), and the third factor consisted of five approach-worded self-image goals (both likable and competent). The second and the third factors that comprised approach and avoidance-worded self-image goals were more strongly correlated with each other (*r* = 0.55) than they were with the first compassionate goals factor (*r*s = 0.33 and 0.34).

**Table 2:** Summary of exploratory factor analysis results for the 18-Item compassionate and self-image goals measure using principal axis factoring and promax rotation with combined American and Japanese samples in study 1 (*N* = 1149).

	Factor loadings				
Item	1	2	3	4	*h*^2^
Have compassion for others’ mistakes and weaknesses.	**0.74**				0.53
Understand the other person’s feelings.	**0.72**				0.57
Be supportive of others.	**0.67**	–0.21			0.55
Avoid taking away others’ opportunity for growth.	**0.67**			–0.24	0.38
Avoid doing anything that would be harmful to others.	**0.63**	0.24			0.48
Avoid neglecting your relationship with others.	**0.52**				0.33
Avoid being embarrassed.		**0.79**			0.55
Avoid looking like a failure.		**0.73**			0.58
Avoid being exposed as wrong.		**0.60**			0.45
Avoid showing your unlikable side.		**0.58**		0.22	0.45
Avoid being disliked.		**0.56**		0.34	0.54
Avoid showing your weaknesses.		**0.50**	0.22		0.36
Prove your competence to others.			**0.68**		0.53
Get others to recognize or acknowledge your intelligence.			**0.68**		0.52
Appear successful.			**0.64**		0.51
Get others to think that you are nice.				**0.74**	0.65
Get others to think that you are kind.				**0.65**	0.62
Be seen as a kind person.	0.28			**0.61**	0.58
Eigenvalues	5.72	2.80	1.60	0.96	
% of variance	31.79	15.34	8.90	5.34	

*Factor loadings under 0.20 are suppressed. Bold numbers indicate highest loadings.*

When a four-factor solution was specified, the first factor comprised the approach and avoidance-worded compassionate goals, the second factor comprised the avoidance-worded liking and competent self-image goals, the third factor comprised the approach-worded competent self-image goals, and the fourth factor the approach-worded likable self-image goals.^3^ The four-factor solution explained 61.6% of total variance. As expected, the second, third, and fourth factors that consisted of self-image goals were highly correlated with each other (0.41 < *r*s < 0.52). The first factor, consisting of compassionate goals, correlated with the fourth factor (i.e., approach-worded likable self-image goals; *r* = 0.50) and moderately with the second (i.e., avoidance-worded likable and competent self-image goals; *r* = 0.22), but less so with the third (i.e., approach-worded competent self-image goals; *r* = 0.09). When a two-factor solution was specified, the self-image goals items comprised the first factor (>0.54) and the compassionate goals items loaded on the second factor (>0.53) except for one approach-worded likable self-image goals (“be seen as a kind person”) which had a loading of 0.30 on the first self-image goals factor and a loading on 0.51 on the second compassionate goals factor. The two factors explained 47.3% of variance and were moderately correlated (*r* = 0.38).

From the EFAs of the 18-item scale, we can conclude that Americans and Japanese: (a) distinguish compassionate goals from self-image goals, (b) think of all the avoidance-worded self-image goals items as one category, without distinguishing the avoidance-worded likable self-image goals and avoidance-worded competent self-image goals, and (c) can distinguish the approach-worded likable self-image goals from the approach-worded competent self-image goals. The reliabilities and the means of the subscales are shown in Table 3.

**Table 3:** Reliabilities, means, and standard deviations of compassionate and self-image goals measures by country (studies 1, 2, and 3).

	Study 1						Study 2						Study 3^a^		
	United States (*n* = 569)			Japan (*n* = 580)			United States (*n* = 550)			Japan (*n* = 897)			Japan (*n* = 274)		
	α	*M*	*SD*	α	*M*	*SD*	α	*M*	*SD*	α	*M*	*SD*	α	*M*	*SD*
Compassionate goals (12 items)	0.81	3.80	0.74	0.82	3.47	0.69	0.82	3.88	0.74	0.82	3.49	0.70	0.83	3.43	0.69
Self-image goals (12 items)	0.88	3.24	0.76	0.85	3.09	0.62	0.89	3.32	0.77	0.84	3.07	0.60	0.88	3.04	0.67
Approach-worded self-image goals (6 items)	0.81	3.28	0.79	0.82	2.89	0.72	0.81	3.41	0.79	0.81	2.85	0.71	0.85	2.81	0.78
Avoidance-worded self-image goals (6 items)	0.86	3.08	0.92	0.76	3.21	0.67	0.85	3.13	0.90	0.75	3.24	0.65	0.78	3.27	0.69
Competent self-image goals (6 items)	0.81	3.07	0.84	0.73	2.89	0.66	0.85	3.17	0.88	0.73	2.88	0.66	0.82	2.83	0.74
Likable self-image goals (6 items)	0.82	3.41	0.83	0.80	3.28	0.71	0.82	3.47	0.81	0.79	3.26	0.68	0.84	3.26	0.75
Approach-worded competent self-image goals (3 items)	0.75	3.09	0.91	0.76	2.65	0.82	0.76	3.20	0.95	0.78	2.60	0.84	0.83	2.57	0.89
Approach-worded likable self-image goals (3 items)	0.79	3.48	0.91	0.80	3.14	0.83	0.80	3.62	0.90	0.80	3.09	0.82	0.85	3.05	0.90

*^*a*^The American sample in Study 3 was the same as in Study 2.*

## Study 2

Study 2 used a multi-group confirmatory factor analysis (CFA) to examine the equivalence of factor structure and factor loadings across cultures with a new sample of American and Japanese participants. Specifically, Study 2 tested for configural invariance (i.e., whether the same items are associated with the same factor in both cultures), metric invariance (i.e., whether the items had equal factor loadings across cultures), and scalar invariance (i.e., whether the intercepts are identical across cultures).

### Methods

#### Participants

A new sample of 709 American participants were recruited through Amazon’s MTurk. Twenty-two participants who failed the attention check question and 89 participants who had a 0 variance on at least one of the five pages containing the goals items were excluded from the dataset. An additional 48 participants were deleted form the dataset because multi-group CFA cannot be performed on data with missing values, leaving a final sample of 550 participants (41.5% female). Their ages ranged from 19 to 79 with a mean of 35.80 (*SD* = 11.54). Similar to Study 1, the majority of participants (94.0%) had lived in the United States for their entire life. Most participants had all four grandparents born in the United States (61.3%), while 12.0% had no grandparents born in the United States. Six participants indicated having lived in Japan, the longest being 5 years. The majority (77.1%) were European or White, 12.3% African American or Black, 5.8% Asian, 6.7% Latino or Hispanic, and 6.5% indicated other ethnicities. In the United States, the consent form was displayed on the first page of the survey.

The Japanese data consisted of the second dataset from Study 1 (*n* = 1134). Two-hundred and thirty-seven participants had one or more missing values in their goals items and were excluded from multi-group CFA. The final Japanese sample consisted of 897 participants (46.8% female), ranging in age from 20 to 77 (*M* = 37.5, *SD* = 12.20).

#### Measures

American and Japanese participants completed the same 50-item compassionate and self-image goals scale as in Study 1. American participants also completed measures assessing interdependence, which will be described in Study 3. AMOS v23 was used in all analyses.

### Results and Discussion

The model had four correlated factors: compassionate goals with 6 indicators, avoidance-worded self-image goals with 6 indicators, approach-worded competent self-image goals with 3 indicators, and approach-worded likable self-image goals with 3 indicators. To test configural invariance, the correlated four-factor model was fitted simultaneously to the American and Japanese data. Metric invariance was tested by further constraining the factor loadings to be equal across cultures. Scalar invariance was tested by further constraining the intercept of the factors to be equal across cultures. As shown in Table 4, the configural invariance model had adequate fit, as indicated by a χ^2^/df under 5.0, CFI greater than 0.90, and RMSEA under 0.08 (van de Schoot et al., 2012). Configural invariance indicates that both Americans and Japanese distinguish compassionate goals, avoidance-worded self-image goals, approach-worded likable self-image goals, and approach-worded competent self-image goals and that the same items are associated with each factor.

**Table 4:** Goodness-of-fit indicators of the four-factor models in multi-group confirmatory analysis that compared United States (*n* = 550) and Japanese Samples (*n* = 897) in Study 2 and United States (*n* = 550) and Japanese Samples (*n* = 250) in Study 3.

Models	χ ^2^	*df*	χ ^2^/*df*	CFI	RMSEA
**Study 2**					
Configural invariance model	1133.69	258	4.394	0.909	0.048
Metric invariance model	1199.21	272	4.409	0.903 (Δ 0.006)	0.049 (Δ 0.001)
Scalar invariance model	1797.32	290	6.198	0.843 (Δ 0.060)	0.060 (Δ 0.011)
Configural invariance of three-factor models that distinguished approach and avoidance-worded self-image goals	1936.11	264	7.33	0.826	0.066
Configural invariance of three-factor models that distinguished competent and likable self-image goals	1766.9	264	6.69	0.844	0.063
Configural invariance of two-factor models with compassionate goals and self-image goals	2353.8	268	8.78	0.783	0.073
**Study 3**					
Configural invariance model	741.62	258	2.874	0.919	0.048
Metric invariance model	774.50	272	2.847	0.916 (Δ 0.003)	0.048 (Δ 0.00)
Scalar invariance model	1140.66	290	2.933	0.857 (Δ 0.059)	0.061 (Δ 0.013)

Constraining the factor loadings to be identical across cultures (i.e., metric invariance) increased χ^2^/df from 4.394 to 4.409, decreased CFI from 0.909 to 0.903, and increased RMSEA from 0.048 to 0.049 but the model continued to show adequate fit. Furthermore, the results showed that the decrease in CFI was 0.006 and the increase in RMSEA was 0.001, meeting the criteria for invariance set by Chen (2007) and Cheung and Rensvold (2002) who suggested that one could assume invariance if the decrease in CFI was equal or smaller than 0.01 and the increase in RMSEA was smaller than 0.015. However, further constraining the intercepts to be equal across cultures considerably decreased the model fit: χ^2^/df increased to 6.198, CFI decreased to 0.843, and RMSEA increased to 0.060. Although the increase in RMSEA (Δ 0.011) was under the criterion of 0.015, the decrease in CFI (Δ 0.06) was larger than 0.01, indicating that one cannot assume scalar invariance. In conclusion, given the metric invariance, one can assume equivalence in the unit of measurement, and can thus compare relationships across cultures. However, given the lack scalar invariance, one needs to be cautious when interpreting mean differences across cultures^4^.

Next, three alternative models were tested to see if the correlated four-factor model had the best fit across cultures. The first alternative model consisted of a correlated three-factor model with compassionate goals, approach-worded self-image goals, and avoidance-worded self-image goals, which was suggested in the first EFA of Study 1. The second alternative model consisted of a correlated three-factor model with compassionate goals, likable self-image goals (both approach- and avoidance-worded), and competent self-image goals (both approach- and avoidance-worded). The third alternative model consisted of a correlated two-factor model that distinguished compassionate goals from self-image goals. As displayed in Table 4, none of the alternative models showed configural invariance, as indicated by χ^2^/df exceeding 5.0 (7.33, 6.69, and 8.78, respectively) and CFI under 0.90 (0.826, 0.844, and 0.783), although RMSEA was still under 0.08 (0.066, 0.063, and 0.073). These results confirmed that the correlated four-factor model was the best and only model that adequately captured both American and Japanese data.

Table 3 shows the reliabilities and the means of the subscales.^5^ The reliabilities obtained in Study 2 were comparable to those from Study 1 and were in the acceptable range (0.73 to 0.89). Studies 1 and 2 together established that the 18-item compassionate and self-image goals scale had a culturally invariant four-factor structure, culturally invariant factor loadings, and good reliabilities.

## Study 3

Study 3 examined the conceptual distinction between compassionate goals and interdependence in the United States and Japan. To do so, it tested whether items from various interdependence scales and compassionate goals items loaded on separate factors when subjected to an exploratory factor analysis. Study 3 also examined the correlations between various measures of interdependence and compassionate, likable self-image, and competent self-image goals in the United States and Japan. Compassionate goals and likable self-image goals were expected to correlate positively but only moderately with various measures of interdependence in both the United States and Japan. Moreover, interdependence was expected to correlate more strongly with likable self-image goals than with competent self-image goals.

### Methods

#### Participants

The American sample consisted of the same 598 American participants (41.8% female) from Study 2 who passed the attention check and who had more than zero variance on the interpersonal goals scale (mean age 35.6, *SD* = 11.39). Those with missing values were excluded in Study 2 but were included in Study 3. In Japan, four-hundred and seventeen participants were recruited through a web survey company (Marketing Applications Inc.). Of these, 279 who failed the attention check question and five who had zero variance on the 18-item compassionate and self-image goals scale were excluded, leaving a final sample of 274 (47.8% female, mean age 37.8, *SD* = 12.20). In Japan, the consent form was displayed in the e-mail sent to recruit potential participants.

#### Measures

American participants completed the 50-item compassionate and self-image goals scale whereas Japanese participants completed the 18-item version of it (see Table 3 for reliabilities). Then, participants completed four interdependence scales.^6^ The first scale was Vignoles et al.’s (2016) independent and interdependent self-construals scale, which captured self-construal along seven dimensions: difference versus similarity (4 items such as “Being different from others makes you feel uncomfortable”), self-containment versus connection to others (3 items such as “If someone in your family is sad, you feel the sadness as if it were your own”), self-direction versus receptiveness to influence (2 items such as “You always ask your family for advice before making a decision”), self-reliance versus dependence on others (4 items such as “You prefer to ask other people for help rather than rely only on yourself”), consistency versus variability (6 items such as “You see yourself differently in different social environments”), self-expression versus harmony (3 items such as “You try to adapt to people around you, even if it means hiding your inner feelings”) and self-interest versus commitment to others (3 items such as “You value good relations with the people close to you more than your personal achievements”). Participants rated how well each of the 25 statements described them on scales ranging from 1 = *not at all* to 5 = *very much*. The independent items were reversed and the means of each subscales were computed. The Japanese translation of the scale was obtained from the authors.

The second scale was the Relational-Interdependent Self-Construal (RISC) scale (Cross et al., 2000), which measured the degree to which people felt close and committed to their important relationships. Participants rated 11 items such as “My close relationships are an important reflection of who I am” on scales from 1 = *strongly disagree* to 5 = *strongly agree*. A back-translation method was used to generate the Japanese version of the scale.

The third scale was the Inclusion of Other in the Self (IOS) scale (Aron et al., 1992). Participants were given pictures of two circles that overlapped at various degree and were asked to select one picture out of seven that best described their relationship with (a) their romantic partner, (b) their family, (c) their best friend, (d) their community, and (e) others in general. Those who were currently not in a relationship were instructed to skip the first question. The average of the five items was used to indicate the degree of overlap between the self and others, the higher value indicating greater overlap.

The fourth scale was the Self-Construal Scale by Singelis (1994), a 24-item scale that measures independent and interdependent self-construals. A Japanese translation of the scale (Heine, 1996) was used in Japan. Participants rated 12 items that measured independent self-construal (e.g., “I enjoy being unique and different from others in many respects.”) and 12 items that measured interdependent self-construal (e.g., “I have respect for the authority figures with whom I interact.”). As suggested by Singelis, one item (“I feel comfortable using someone’s first name soon after I meet them, even when they are much older than I am”) was replaced with (“I can talk openly with a person who I meet for the first time, even when this person is much older than I am”).

### Results and Discussion

#### Equivalence of the Goals Scale

First, a multi-group CFA was conducted to check the measurement equivalence of the 18-item compassionate and self-image goals scale. The American sample consisted of 550 as in Study 2. The Japanese sample consisted of 250 participants because 24 participants who had one or more missing values in the 18-item goals scale were excluded from the analysis. As shown at the bottom of Table 4, the configural invariance model had an acceptable fit, χ^2^/*df* = 2.874, CFI = 0.919, RMSEA = 0.048. The metric invariance model resulted in a 0.003 decrease in CFI, and no increase in RMSEA, supporting metric invariance. The scalar invariance model had poor fit, χ^2^/*df* = 2.933, CFI = 0.857, RMSEA = 0.061. The decrease in CFI (Δ 0.059) was larger than the criterion 0.01; although the increase in RMSEA (Δ 0.013) was smaller than the criterion 0.015, overall, one could not assume scalar invariance (Cheung and Rensvold, 2002; Chen, 2007). Consistent with Study 2, these results confirmed that the 18-item goals scale had similar factor structure and factor loadings in the United States and Japan, making it possible to compare correlations across cultures.

#### Compassionate Goals and Interdependent Measures

Next, EFAs were conducted with the six compassionate goals items and the interdependent self-construals items from each scale, separately for each culture, to see whether Americans (*n* = 598) and Japanese (*n* = 274) distinguished compassionate goals from interdependence. In all analyses, a principal axis method was used for extraction with promax rotation. The number of factors was determined according to the decrement in eigen values. The results are summarized in Table 5.

**Table 5:** Summary of exploratory factor analyses with compassionate goals and interdependence items by country (study 3).

Scales	Country	Extracted factors (% of variance)	Compassionate items loadings on the compassionate factor	Other goals items with primary loadings on the compassionate factor	Compassionate items with cross-loadings (> 0.20) on other factors	Other goals items with cross-loadings (>0.20) on the compassionate factor
Vignoles Interdependence	United States	6 (59.9%)	All 6 items (>0.59)	“You value good relations with the people close to you more than your personal achievements” (0.37)	–	“Your own success is very important to you, even if it disrupts your friendships (*r*)” (0.29)
						“You value personal achievements more than good relations with the people close to you (*r*)” (0.30)
						“You prefer to do what you want without letting your family influence you (*r*)” (-0.24)
	Japan	5 (61.3%)	All 6 items (>0.54)	–	–	“Your own success is very important to you, even if it disrupts your friendships (*r*)” (0.34)
						“You feel uncomfortable in situations where you have to rely only on yourself” (-0.24)
						“You see yourself differently in different social environments” (0.25)
Relational Interdependence	United States	3 (60.7%)	All 6 items (>0.54)	–	–	“I usually feel a strong sense of pride when someone close to me has an important accomplishment” (0.29)
						“If a person hurts someone close to me, I feel hurt as well” (0.35)
	Japan	3 (57.3%)	All 6 items (>0.53)	–	“Avoid neglecting your relationship with others” (0.21)	–
Inclusion of Others in Self	United States	2 (54.6%)	All 6 items (>0.52)	–	–	IOS with romantic partner (0.21)
	Japan	3 (63.5%)	All 6 items (>0.58)	–	–	–
Singelis Interdependence	United States	2 (42.8%%)	All 6 items (>0.54)	“I respect people who are modest about themselves” (0.32)	–	–
	Japan	2 (41.9%)	All 6 items (>0.51)	“I respect people who are modest about themselves” (0.38)	“Avoid doing anything that would be harmful to others” (-0.29)	“I often have the feeling that my relationships with others are more important than my own accomplishments” (0.21)
						“It is important for me to maintain harmony within my group” (0.30)
					“Avoid neglecting your relationship with others” (0.21)	
						“It is important to me to respect decisions made by the group” (0.22)

Across all the factor analyses, compassionate goals emerged as a separate factor from factors related to interdependence, indicating that both Americans and Japanese can and do distinguish these two concepts. In both cultures and across all the analyses, the six compassionate goals items loaded primarily on the compassionate goals factor, with loadings greater than 0.51. Two interdependent items also loaded primarily on the compassionate goals factor (“You value good relations with the people close to you more than your personal achievements” and “I respect people who are modest about themselves”), but their loadings were weaker (0.32–0.38) than those of the compassionate goals items, indicating that these items are not the main ingredient of the compassionate goals factor. Some compassionate goals items had cross-loadings greater than | 0.20| on the interdependent factors (for example, in Japan, “Avoid neglecting your relationship with others” showed a cross-loading on the relational interdependence factor and on Singelis’ interdependence) while some interdependent items cross-loaded on the compassionate goals factor [e.g., “Your own success is very important to you, even if it disrupts your friendships (*reversed*)”], suggesting that some aspects of interdependence may be conceptually closer to compassionate goals. However, these cross-loadings were relatively low (| 0.21|–| 0.34|), attesting to the conceptual distinction between compassionate goals and interdependence.

#### Correlations Between Interdependence and Interpersonal Goals

Correlations between various indices of interdependence and interpersonal goals are displayed in Table 6. Because compassionate goals correlated with approach-worded likable self-image goals (0.47 in the United States and 0.44 in Japan) and avoidance-worded self-image goals (0.18 in the United States and 0.43 in Japan), partial correlations were also computed. Compassionate goals were regressed on the composite of all 12 self-image goals items and the residuals were saved as a “pure” measure of compassionate goals. Similarly, self-image goals were regressed on compassionate goals and the residuals were saved as “pure” measures of likable, competent, and avoidance-worded self-image goals. There correlations between these “pure” measures and indices of interdependence are shown in the right half of Table 6^7^.

**Table 6:** Correlations and partial correlations between interpersonal goals and measures of interdependence by country (study 3).

United States (*n* = 598)	correlations						partial correlations			
	Compassio-nate goals	SI goals	Likable SI goals	Competent SI goals	Avoidance-worded SI goals	Compassio-nate goals^b^	SI goals^c^	Likable SI goals^c^	Competent SI goals^c^	Avoidance-worded SI goals^c^
Similarity^a^	–0.127**	0.165**	0.060	0.075	0.237**	–0.184**	0.212**	0.136**	0.086*	0.264**
Variability^a^	–0.175**	0.287**	0.135**	0.225**	0.330**	–0.273**	0.356**	0.246**	0.240**	0.368**
Connection to others^a^	0.413**	0.129**	0.267**	0.044	0.026	0.393**	0.006	0.082*	0.009	–0.049
Dependence on others^a^	–0.020	0.153**	0.116**	0.151**	0.142**	–0.069	0.167**	0.142**	0.153**	0.148**
Commitment to others^a^	0.417**	–0.154**	0.094*	–0.298**	–0.190**	0.485**	–0.292**	–0.116**	–0.334**	–0.270**
Receptiveness to influence^a^	0.059	0.183**	0.140**	0.216**	0.140**	0.005	0.173**	0.127**	0.212**	0.132**
Harmony^a^	0.057	0.141**	0.172**	–0.013	0.156**	0.014	0.130**	0.166**	0.017	0.149**
Relational Interdependence	0.471**	0.254**	0.368**	0.171**	0.124**	0.414**	0.119**	0.166**	0.132**	0.039
Singelis Independence	0.250**	0.018	0.087*	0.116**	–0.092*	0.256**	–0.059	–0.035	0.095*	–0.139**
Singelis Interdependence	0.384**	0.427**	0.450**	0.279**	0.334**	0.269**	0.328**	0.305**	0.247**	0.268**
Inclusion of Others in Self	0.191**	0.151**	0.173**	0.168**	0.078	0.153**	0.099*	0.095*	0.153**	0.044
**Japan (*n* = 274)**		
Similarity^a^	0.017	0.021	0.050	–0.132*	0.092	0.009	0.016	0.047	–0.135*	0.094
Variability^a^	0.028	0.135*	0.082	0.011	0.198**	–0.090	0.160**	0.105	0.013	0.233**
Connection to others^a^	0.317**	0.136*	0.107	–0.006	0.178**	0.287**	0.010	–0.037	–0.037	0.047
Dependence on others^a^	–0.125*	–0.082	–0.090	–0.046	–0.080	–0.100	–0.035	–0.039	–0.034	–0.030
Commitment to others^a^	0.291**	–0.175**	–0.012	–0.360**	–0.104	0.394**	–0.319**	–0.155**	–0.390**	–0.253**
Receptiveness to influence^a^	0.177**	0.097	0.151*	–0.012	0.095	0.150*	0.029	0.081	–0.029	0.022
Harmony^a^	0.098	–0.065	0.039	–0.266**	0.011	0.135*	–0.114	–0.005	–0.277**	–0.034
Relational Interdependence	0.464**	0.308**	0.299**	0.179**	0.275**	0.373**	0.133*	0.104	0.134*	0.084
Singelis Independence	0.403**	0.202**	0.145*	0.249**	0.136*	0.352**	0.044	–0.036	0.210**	–0.041
Singelis Interdependence	0.562**	0.340**	0.330**	0.181**	0.313**	0.465**	0.125*	0.092	0.127*	0.079
Inclusion of Others in Self	0.283**	0.121*	0.074	0.076	0.130*	0.256**	0.008	–0.056	0.048	0.010

*^*a*^Subscales of Vignoles et al.’s (2016) scale. ^*b*^Residuals after controlling for the 12-item self-image goals. ^c^Residuals after controlling for compassionate goals. ^∗∗∗^*p* < 0.001; ^∗∗^*p* <0.01; ^∗^*p* <0.05.*

In both cultures, compassionate goals (controlling for self-image goals) correlated positively and moderately with various indices of interdependence, including connection to others (*pr*_US_ = 0.39; *pr*_Jp_ = 0.29), commitment to others (*pr*_US_ = 0.49; *pr*_Jp_ = 0.39), relational interdependent self-construal (*pr*_US_ = 0.41.; *pr*_Jp_ = 0.37), Singelis’ interdependence (*pr*_US_ = 0.27; *pr*_Jp_ = 0.47), and inclusion of others in self (*pr*_US_ = 0.15; *pr*_Jp_ = 0.26). In Japan, compassionate goals also correlated with receptiveness to influence (0.26) and harmony (0.14). As expected, these modest correlations corroborated the idea that the pursuit of compassionate goals is related to, but not identical to interdependence.

Surprisingly, compassionate goals were negatively correlated with some aspects of interdependence. For example, in the United States, compassionate goals were negatively associated with similarity (-0.18) and variability (-0.27). Moreover, in both cultures, compassionate goals were positively correlated with Singelis’ independence (*pr*_US_ = 0.26; *pr*_Jp_ = 0.35). These findings suggest that compassionate goals can be pursued via both independent and interdependent selves.

Likable self-image goals were also positively and moderately correlated with indices of interdependence but they correlated with aspects of interdependence that were different from those associated with compassionate goals. Unlike compassionate goals, which correlated with measures reflecting close relationships with others (e.g., connection with others, commitment to others, relational interdependent self-construal, and inclusion of others in self), likable self-image goals were associated with measures related to conformity: In the United States, likable self-image goals (controlling for compassionate goals) were associated with similarity (0.14), variability (0.25), connection to others (0.08), dependence on others (0.14), receptiveness to influence (0.13), harmony (0.17), relational interdependence (0.17), Singelis’ interdependence (0.31), and inclusion of others in self (0.10). None of these associations were significant in Japan. Additionally, in stark contrast to compassionate goals, likable self-image goals were negatively associated with commitment to others in both the United States (-0.12) and Japan (-0.16). That these goals correlated with different aspects of interdependence confirmed that although compassionate and likable self-image goals may both involve prosocial tendencies (e.g., be supportive of others and be seen as a kind person), they are distinct constructs. The results also suggest that being interdependent involves pursuing not only compassionate goals but also likable self-image goals.

In the United States, the correlations for avoidance-worded self-image goals and competent self-image goals were similar to those for likable self-image goals, presumably due to the high correlations among the self-image goals (0.46 < *r*s < 0.67). As with likable self-image goals, both avoidance-worded self-image goals and competent self-image goals (controlling for compassionate goals) were associated with similarity (0.26 and 0.09), variability (0.37 and 0.24), dependence on others (0.15 and 0.15), receptiveness to influence (0.13 and 0.21), and Singelis’ interdependence (0.27 and 0.25). Competent self-image goals (but not avoidance-worded self-image goals) correlated with relational interdependence (0.13) and inclusion of others in self (0.15); avoidance-worded self-image goals (but not competent self-image goals) correlated with harmony (0.15). As with likable self-image goals, both goals were negatively associated with commitment to others (-0.27 and -0.33).

Taken together, these correlations show that in the United States, (a) aspects of interdependence that reflected conformity were more closely associated with likable, competent, and avoidance-worded self-image goals than with compassionate goals and (b) aspects of interdependence that reflected connection to others were more associated with compassionate goals than with self-image goals. With self-image goals, people seem to adjust their behaviors to the surrounding norms, but may feel less connected to others. Although we expected interdependence to correlate more strongly with likable self-image goals than with competent self-image goals, the results were mixed: Some aspects of interdependence (i.e., similarity, connection to others, harmony, relational interdependent self-construal, and Singelis’ interdependence) correlated more strongly with likable self-image goals while others (i.e., dependence on others, receptiveness to influence, and inclusion of others in self) correlated more strongly with competent self-image goals. People with an interdependent orientation were more concerned about appearing likable than those with an independent orientation, but they were no more likely to pursue likable self-image goals than competent self-image goals.

In Japan, very few significant correlations emerged. As in the United States, all three self-image goals correlated negatively with commitment to others (-0.15 < *pr*s < -0.39), avoidance self-image goals correlated positively with variability (0.23), and competent self-image goals correlated with relational interdependent self-construal (0.13), Singelis’ independence (0.21) and interdependence (0.13). The weak associations between self-image goals and interdependence in Japan suggests that the Japanese may not necessarily behave in an interdependent way to project likable or avoid projecting dreaded self-images. Interestingly, competent self-image goals correlated negatively with harmony (-0.28) and similarity (-0.14) in Japan but not in the United States. The negative association between competent self-image goals on the one hand and similarity and harmony on the other may reflect the Japanese preference for self-criticism over self-enhancement (Kitayama et al., 1997; Heine et al., 1999). Trying to appear competent in a Japanese context may be incompatible with maintaining group harmony and ensuring similarity with others.

## General Discussion

The purpose of the present research was to examine how culture relates to different interpersonal goals in the United States and Japan. Although cultures vary in the ideal self-images people are likely to pursue, the extent to which cultures emphasize the pursuit of compassionate goals remained unclear. Our research provides empirical evidence that compassionate goals are related but distinct from interdependence. In Study 1, the 18-item scale showed a clear four-factor structure that distinguished (a) compassionate goals, (b) approach-worded likable self-image goals, (c) approach-worded competent self-image goals, and (d) avoidance-worded self-image goals. Study 2 confirmed the equivalence of the four-factor structure and the equivalence of factor loadings in the United States and Japan. Finally, Study 3 showed that the items of compassionate goals and those of various measures of interdependence loaded into separate factors with low cross-loadings, corroborating the idea that Americans and Japanese distinguish compassionate goals from interdependence. Study 3 further found that the indices of interdependence showed moderately positive correlations with compassionate goals as well as with likable, competent, and avoidant self-image goals, indicating that the pursuit of compassionate goals is not the sole feature of interdependence.

### Interdependence and Compassionate Goals

Are compassionate goals a defining feature of an interdependent self? Compassion and sympathy may be an important mechanism through which people maintain interdependence (Kitayama and Markus, 2000) but the current findings suggest that the pursuit of compassionate goals is not a defining feature of interdependence. First, across all exploratory factor analyses in Study 3, compassionate goals emerged as a separate factor from interdependence, with low cross-loadings. This is evidence that both Americans and Japanese distinguish these two concepts and that one is not a component of the other. Second, when controlling for self-image goals, the associations between compassionate goals and the various indices of interdependence ranged from -0.27 to 0.49 in the United States and from -0.10 to 0.47 in Japan. These moderate associations show that interdependence and compassionate goals are related but not identical.

Which aspects of interdependence are conceptually closer to compassionate goals? In the exploratory factor analyses, some compassionate goals items cross-loaded on the interdependent factors while some interdependent items cross-loaded on the compassionate goals factor. Six out of the 12 interdependent items that also loaded on compassionate goals factor (e.g., “You value good relations with the people close to you more than your personal achievements,” “I often have the feeling that my relationships are more important than my own accomplishments”) and the two compassionate goals items that cross-loaded on the interdependent factor (“Avoid doing anything that would be harmful to others” and “Avoid neglecting your relationship with others”) were about valuing relationships and not sacrificing relationships for the sake of personal gain. These items seem to reflect a nonzero-sum belief about relationships—the belief that “what is good for one person in a relationship is (or can be) good for both, and that it is possible for both relationship partners to get what they need” (Crocker et al., 2017, p. 59). People with compassionate goals tend to have a nonzero-sum view of relationships (Crocker and Canevello, 2008; Crocker et al., 2017), and therefore, the pursuit of compassionate goals may show conceptual overlap with aspects of interdependence that concern not sacrificing relationship for the sake of personal gains.

The feeling of connection with others was another facet of interdependence that was conceptually close to compassionate goals. Compassionate goals (when controlling for self-image goals) were positively associated with measures of interdependence that reflected close relationships with others (e.g., connection with others, commitment to others, relational interdependent self-construal, and inclusion of others in self) but not with measures related to conformity (similarity, variability, dependence on others in both cultures, and receptiveness to influence and harmony in the United States). These results are consistent with past research which found that compassionate goals predicted increased relational interdependent self-construal, feelings of connection, relationship closeness, and commitment over time (Canevello and Crocker, 2010; Jiang et al., 2017). They were also in line with longitudinal evidence that compassionate goals predict feeling more connected, cooperative, loving, empathic, peaceful, clear, present, and engaged (Canevello and Crocker, 2017). Compassionate goals may encourage feeling connected to others, while the perception of close interdependent relationships may, in turn, facilitate the endorsement of compassionate goals.

Although compassionate goals were positively correlated with some aspects of interdependence, compassionate goals were also positively correlated with independence. In fact, compassionate goals (controlling for self-image goals) were associated with Singelis’ interdependence (0.27 in the United States and 0.47 in Japan) as much as with Singelis’ independence (0.26 in the United States and 0.35 in Japan). In the United States, compassionate goals also showed negative association with similarity and variability (i.e., compassionate goals were associated with greater tendency to see oneself as being different from others and a greater tendency to behave consistently across situations). These findings further attest that compassionate goals are not a unique marker of interdependence. The pursuit of compassionate goals may require some assertiveness and autonomy as people commit to supporting others’ well-being (Niiya, 2016).

The idea of interdependence is often associated with warm and caring relationships among members of the group whereas independence is often associated with self-centered, task-oriented, or instrumental relationships, but our research implies that being interdependent does not necessarily encourage the pursuit of compassionate goals. One could be interdependent and conform to a group decision or vary behaviors depending on contexts to satisfy others’ needs (e.g., improve a group’s performance) or to satisfy one’s own need (i.e., get recognition). At times, pursuing compassionate goals may even require people to confront others and receive criticism from them if doing so benefits the group. This research attests to the importance of differentiating the cultural value and behavioral norms of interdependence from the interpersonal goals people pursue in their everyday relationships.

### Interdependence and Self-Image Goals

Are people with an interdependent self more likely to pursue self-image goals? Various measures of interdependence correlated positively with all three types of self-image goals, suggesting that being interdependent also involves the goals of projecting desirable and avoiding undesirable self-images. Given the importance of “fitting in” to the surrounding social context (Markus and Kitayama, 2010), it is not surprising that interdependence was associated with seeking recognition and acceptance through self-image goals. Interestingly, self-image goals and compassionate goals were each associated with different aspects of interdependence. In contrast to compassionate goals, which were mostly associated with feeling connected to others, in the United States, self-image goals were associated with measures of conformity, such as dependence on others, receptiveness to influence, harmony, similarity to others, and variability of the self across situations, and with reduced commitment in both cultures. People who want to project a desirable self-image or who want to avoid a dreaded self-image may be more inclined to follow norms, adjust themselves to the surrounding context, and hide their inner feelings to gain acceptance from others, but consequently, they may suffer from reduced feeling of connection with others.

Unexpectedly, interdependence did not always correlate more strongly with likable self-image goals than with competent self-image goals. Likable self-image goals reflected a more collectivistic value (Wojciszke, 1997; Judd et al., 2005), and hence, should have been preferred among those with interdependent selves. However, in the United States, while some aspects of interdependence (i.e., similarity, connection to others, harmony, relational interdependent self-construal, and Singelis’ interdependence) correlated more strongly with likable self-image goals, others (i.e., dependence on others, receptiveness to influence, and inclusion of others in self) correlated more strongly with competent self-image goals. In the United States, both appearing competent and appearing likable may serve the goals to obtain a favorable evaluation of the self, which may help maintain interdependence, while concerns for interdependence may motivate people to seek acceptance by projecting both likable and competent images of the self. In contrast, in Japan, except for the negative associations between competent self-image goals and concern for harmony, commitment to others, and similarity with others, none of the correlations between self-image goals and interdependence was significant. In a culture that encourages self-criticism over self-enhancement (Kitayama et al., 1997; Heine et al., 1999), only those who are low in interdependence may seek recognition by demonstrating their competence. The lack of association between interdependence and self-image goals in Japan was unexpected—one possible interpretation may be that the Japanese tendency to pursue self-image goals may vary depending on the situations more so than the Americans such that when these goals are measured as a trait (i.e., goals people generally pursue across different relationships), they may not be sensitive enough to capture the associations with interdependence.

### Implications of the New Scale

This research also contributed to the establishment of a compassionate and self-image goals scale that shows measurement invariance across the United States and Japan. This new scale represents an improvement over existing scales, which also showed measurement invariance in the United States and Japan (Niiya et al., 2013) and in Poland (Kuncewicz et al., 2015), on four grounds: First, in contrast to previous scales which did not make a clear distinction between competent and likable self-image goals, the new scale distinguishes likable vs. competent self-image goals. This distinction may be important in future cross-cultural research aiming to understand how culture moderates the influence of self-image goals in shaping relationships and well-being, especially because likable self-image goals may be more central to collectivistic cultures and competent self-image goals more central to individualistic cultures. The distinction between likable and competent self-image goals may also be important in elucidating whether the content of self-image goals moderates the negative impact of self-image goals on relationships and well-being (e.g., Crocker et al., 2010; Canevello and Crocker, 2011; Canevello et al., 2013). Second, the new scale includes items that were generated in Japan (Niiya, 2016) as well as the United States (Canevello and Crocker, unpublished), rather than importing and translating items from a single culture (Niiya et al., 2013). This approach ensures that the items better reflect the interpersonal goals commonly experienced and understood in each culture (Cheung et al., 2011). Third, the new scale has equal numbers of approach and avoidance-worded items, making it possible for future research to examine whether the goals to appear likable or competent and the goals to avoid projecting negative self-images have differential effects on behaviors. Finally, the new scale has considerably improved reliabilities (0.73 < αs < 0.89) over previous scales (0.55 < αs < 0.72).

### Future Directions

This research has some limitations that need to be addressed in future research. We demonstrated cultural invariance of the 18-item compassionate and self-image goals scale in the United States and Japan, two countries that have often represented the independent and the interdependent cultures in cultural psychology (e.g., Markus and Kitayama, 2010). It is noteworthy that we found similarities in factor structures, loadings, and correlations between these two cultures that differ in so many aspects (language, religion, educational system, etc.). Nonetheless, interdependence in Japan may not be identical to interdependence in other East Asian countries, or interdependence among ethnic minorities or working class population in the United States. The amount of overlap between the pursuit of compassionate goals and interdependence may also vary by culture.

Moreover, the cross-sectional nature of the data does not allow inference about the causal direction of the association between interdependence and interpersonal goals. Causal associations are likely complex, because cultures encourage certain goals in individuals, while individuals’ goals shape behaviors and cultures (Markus and Kitayama, 2010). To disentangle this cycle of mutual constitution, researchers need to manipulate the goals or prime cultures. They could also use a situational sampling methodology and observe the interplay between cultures and interpersonal goals on relationships and well-being outcomes within different cultural contexts. We hope the culture invariant measure of compassionate and self-image goals scale can serve as a useful tool for researchers toward this end.

Compassion is an important quality that is universally valued (Goetz et al., 2010). Compassion has often been assumed to be a defining characteristic of an interdependent self but this paper showed that independent and interdependent cultures can each shape compassionate goals, and that compassionate goals can be expressed through both independent and interdependent ways of being. That compassionate goals are not a unique product of an interdependent culture implies that people with a strong independent orientation can also pursue compassionate goals. The specific behaviors people use to pursue compassionate goals may still differ between cultures—people from interdependent cultures might show compassion by indirectly attuning to other people’s needs whereas those from independent cultures might be more direct and may show compassion by asking others what they need. Understanding how cultures shape the manifestation of these interpersonal goals would be an important topic for future investigation.

## Ethics Statement

This study was exempt from review by the Stanford Institutional Review Board (Protocol No. 46452) because it met the following criterion: Research involving the use of educational tests (cognitive, diagnostic, aptitude, and achievement), survey procedures, interview procedures, or observation of public behavior UNLESS: (i) information obtained is recorded, such that human subjects can be identified directly or through identifiers linked to the subjects; AND (ii) any disclosure of the human subjects’; responses outside the research could reasonably place subjects at risk of criminal or civil liability or be damaging to the subjects’; financial standing, employability, or reputation.

## Author Contributions

YN and JC conceived and prepared the materials of the studies. YN collected and analyzed the data, and wrote the manuscript in consultation with JC.

## Conflict of Interest Statement

The authors declare that the research was conducted in the absence of any commercial or financial relationships that could be construed as a potential conflict of interest.

## Footnotes

1 Deleting these participants did not change the results.

2 For example, “be supportive of others” which had a loading of 0.67 was chosen over “consider things from the other person’s perspective” which loaded more highly (0.73) on the compassionate goals factor because the former better reflected the concept of compassionate goals. Some likable self-image goals items which also reflected competent self-image goals (e.g., “earn respect of others” or “be seen as a reliable person”) were not included in the shorter scale because the goal of this research was to develop a scale that clearly distinguished likable from competent self-image goals.

3 The same results were obtained even when the American and the Japanese datasets were analyzed separately. EFAs specifying a four-factor solution showed that the same items loaded on the same factor across cultures. Separate EFAs of the 18 items by gender showed the same four-factor structure with similar factor loadings for men and women.

4 A multigroup CFA comparing men and women showed configural (χ^2^= 1110,18, *df* = 258, χ^2^/*df* = 4.303, CFI = 0.913, RMSEA = 0.048), and metric (χ^2^= 1125.29, *df* = 272, χ^2^/*df* = 4.137, CFI = 0.913, RMSEA = 0.047), but no scalar invariance between genders (χ^2^ = 1199.74, *df* = 290, χ^2^/*df* = 4.482, CFI = 0.897, RMSEA = 0.049). Consistent with past research (Crocker and Canevello, 2008), in both Studies 1 and 2, women scored significantly higher than men on compassionate goals (*M*_women_ = 3.74, *M*_men_ = 3.54, *t* = -4.31, *p* < 0.001 in Study 1; *M*_women_ = 3.81, *M*_men_ = 3.50, *t* = -8.33, *p* < 0.001 in Study 2). Also consistent with past research, men and women did not differ in self-image goals (*M*_women_ = 3.17, *M*_men_ = 3.15, *t* = -0.385 in Study 1; *M*_women_ = 3.18, *M*_men_ = 3.16, *t* = -0.645 in Study 2). A closer look showed that in both studies, women were significantly lower than men on competent self-image goals (*M*_women_ = 2.75, *M*_men_ = 2.97, *t* = 4.07, *p* < 0.001 in Study 1; *M*_women_ = 2.73, *M*_men_ = 2.90, *t* = 3.52, *p* < 0.001 in Study 2), but not different from men on avoidance self-image goals (*M*_women_ = 3.19, *M*_men_ = 3.11, *t* = -1.68, in Study 1; *M*_women_ = 3.30, *M*_men_ = 3.25, *t* = -1.39, in Study 2). As for likable self-image goals, gender difference was not significant in Study 1 (*M*_women_ = 3.34, *M*_men_ = 3.28, *t* = -1.14), but significant in Study 2 (*M*_women_ = 3.38, *M*_men_ = 3.22, *t* = -3.26, *p* = 0.001).

5 Americans were higher than the Japanese on compassionate goals, approach-worded likable self-image goals, and approach-worded competent self-image goals but no cultural difference was found for avoidance-worded self-image goals. However, one needs to be careful when interpreting the mean differences as they could reflect genuine cultural differences as well as measurement artifacts. Note also that Americans endorsed the approach-worded self-image goals more than the avoidance-worded self-image goals whereas the reverse was true of the Japanese.

6 American participants also completed other scales not reported in this paper.

7 The correlations between interpersonal goals and interdependence were mostly similar across genders except for the following correlations (controlling for the variance of the other goals): Compassionate goals among women were more strongly associated with variability (*r*_women_ = -0.29, *r*_men_ = -0.16) and dependence (*r*_women_ = -0.19, *r*_men_ = 0.02) whereas compassionate goals among men were more strongly associated with relational interdependence (*r*_women_ = 0.26, *r*_men_ = 0.44) and Singelis’ interdependence (*r*_women_ = 0.22, *r*_men_ = 0.39), suggesting that for women, pursuing compassionate goals meant being less interdependent, whereas for men, it meant being more interdependent. For women, pursuing likable (*r*_women_ = -0.12, *r*_men_ = 0.02) and avoidance self-image goals (*r*_women_ = -0.29, *r*_men_ = 0.02) were more strongly correlated with reduced independence (as measured by Singelis’ scale). For men, pursuing likable self-image goals were associated more strongly with Singelis interdependence (*r*_women_ = 0.13, *r*_men_ = 0.27) whereas pursuing competent self-image goals were associated more strongly with Singelis independence (*r*_women_ = 0.01, *r*_men_ = 0.19).
